# Risk and protective factors for mental health morbidity in a community sample of female-to-male trans-masculine adults

**DOI:** 10.1186/s12888-018-2008-0

**Published:** 2019-01-09

**Authors:** Michal J. McDowell, Jaclyn M. White Hughto, Sari L. Reisner

**Affiliations:** 1 Harvard Medical School, 300 Longwood Ave, Boston, MA USA; 2 Department of Epidemiology, Brown University School of Public Health, Providence, RI USA; 3 The Fenway Institute, Fenway Health, Boston, MA USA; 4 Department of Epidemiology, Harvard T.H. Chan School of Public Health, Boston, MA USA; 5 Division of General Pediatrics, Boston Children’s Hospital, Boston, MA USA

**Keywords:** Transgender, Mental health, Discrimination, Resilience, Violence

## Abstract

**Background:**

Trans-masculine (TM) individuals, who are assigned female sex at birth and identify along the masculine gender spectrum, face mental health disparities relative to cisgender people. Limited research has sought to explore the multi-level risk and protective factors associated with mental health morbidity for TM populations.

**Methods:**

Between August 2015–September 2016, 150 TM adults were enrolled in a one-time biobehavioral health study. A survey assessed socio-demographics, past 12-month everyday discrimination, lifetime intimate partner violence (IPV), resilience (using the Brief Resilience Scale), and other factors. Bivariate and multivariable logistic regression analyses examined associations between participant characteristics and four mental health statuses: post-traumatic stress disorder (PTSD), depression, anxiety, and non-suicidal self-injury (NSSI).

**Results:**

In this sample (76.7% had a binary gender identity, i.e., man or transgender man; 74.7% were white, 70.0% were under age 30 years), 42.2% had PTSD based on past 30-day symptoms; 25.7% had depression based on past 7-day symptoms; 31.1% had anxiety based on past 7-day symptoms; and 31.3% had engaged in NSSI within the past 12-months. Results from multivariable models: 1) PTSD: unemployment, lifetime IPV and past 12-month discrimination were each associated with increased odds of PTSD, while having a partner was associated with the reduced odds of PTSD. 2) Depression: lower educational attainment and past 12-month discrimination were each associated with the increased odds of depression, while greater resilience was associated with the reduced odds of depression. 3) Anxiety: low annual household income and past 12-month discrimination were each associated with the increased odds of anxiety, while resilience was associated with the reduced odds of anxiety. 4) NSSI: past 12-month discrimination was associated with the increased odds of past 12-month NSSI, while higher age and greater resilience was associated with the reduced odds of NSSI (all *p*-values < 0.05).

**Conclusions:**

Unemployment, low income, limited education, everyday discrimination, and violence were risk factors for poor mental health, while being in a relationship, higher age, and personal resilience were protective against mental health morbidity. Findings highlight the need for interventions to address the individual, interpersonal, and societal factors that may be driving poor mental health in this population.

## Background

Trans-masculine (TM) is a term utilized to describe individuals who were assigned a female sex at birth and identify along the masculine gender spectrum, as male, men, transmen, or another diverse gender identity different from their birth sex. About 1.4 million adults in the United States identify as transgender, roughly the population size of Phoenix, the fifth largest city in the country [1, 2]. Mental health research published to date documents worse mental health outcomes in gender minority individuals (i.e., individuals who do not identify as cisgender) as compared to cisgender people. Mental illness is associated with worse quality of life and is a risk factor for suicide [3]. Findings from the Centers for Disease Control and Prevention’s (CDC) Behavioral Risk Factors Surveillance System (BRFSS) survey document a higher prevalence of more days per month of poor mental health in gender minority adults as compared to cisgender adults [4, 5]. Further, previous studies show that the prevalence of depression or depressive symptoms in gender minority adults ranges from 35 to 62%, depending on the measure used and timeframe [6–13], as compared with an estimated 17% lifetime prevalence of major depressive disorder in the United States (U.S.) general population [14].

Literature documenting mental health status for TM individuals specifically or exploring intra-population differences between TM and trans-feminine (TF) people are sparse. One study exploring mental health status in a sample of 155 transgender individuals (52 TM and 103 TF) found TF individuals reported higher scores on anxiety and depression scales compared with TM people [15]. Studies have documented significantly higher prevalence of non-suicidal self-injury (NSSI) in transgender people relative to cisgender people [16], with TM individuals at twice the risk for NSSI compared to trans-feminine (TF) people [15, 17]. Further individual study of populations within the gender minority community is crucial to appropriately tailor supportive resources for these underserved populations. Further understanding of risk factors driving poor mental health among gender minorities and identifying the factors that may protect this population is requisite.

Transgender stigma is a known risk factor for poor mental health for transgender individuals. Research shows that transgender stigma is common [18–21] and impacts transgender individuals directly via victimization (e.g., physical and sexual assault, bullying) as well as indirectly via the internalization of stigma [20, 22]. In one community sample of transgender adults, victimization (including everyday discrimination, bullying, physical assault by family, verbal harassment by family, childhood sexual abuse, and IPV) was shown to be associated with higher depressive symptomology [23], while everyday discrimination was shown to be associated with elevations in posttraumatic stress disorder (PTSD) symptoms [24]. In two studies exploring suicide risk in a population of transgender veterans, authors found that gender-specific discrimination and rejection predicted suicidal ideation [25, 26]. Discrimination in health care settings in the previous year has also been found to be independently associated with an increased risk of adverse emotional and physical symptoms in this population [27]. While stigma is consistently shown to be a driving force behind the heightened poor mental health experienced by transgender individuals overall, little research has focused on the mental health of TM individuals specifically. Thus, there is a need to explore the role of stigma-based victimization in the mental health of TM adults separately.

Research documenting the protective factors for mental health in gender minority adults is limited, as much of the mental health research focuses on risk. Among the limited research on strength and resilience (i.e., recovering, coping, or adapting in the face of adversity) in transgender populations, several individual and interpersonal factors have been documented. Social support, as well as resiliency, are thought to be effective in combating transgender stigma [20, 28, 29]. For example, one study found that transgender social support and community involvement moderated the association between stigma and psychological distress [20, 30]. Another study exploring factors promoting resilience among gender minority individuals found that having higher income, identifying as heterosexual, and having frequent contact with lesbian, gay, bisexual, and transgender peers, were associated with greater resilience (using the Brief Resilience Scale) [29]. Less, however, is known about the impact of discrimination, IPV, and other demographic factors that may enhance risk for mental illness or promote resilience to cope both with life stressors, as well as mental illness.

## Methods

### Aims

Given the gaps in the literature regarding risk and protective factors for mental health morbidity in TM adults in particular, the aims of this study are to: 1) describe the socio-demographic characteristics, discrimination and violence, and resilience and social support experienced by TM adults; and 2) identify risk and protective factors for adverse mental health status in this traditionally under-researched group. These findings can be used to develop interventions that address individual, interpersonal, and societal risk factors, as well as to leverage protective factors to improve mental health status for TM adults.

### Sample

This is a secondary analysis of data collected from 150 TM individuals enrolled in a Boston-based biobehavioral sexual health study between March 2015 and September 2016. Main findings from the sexual health study have been reported elsewhere [31]. Participants were recruited for a single visit through convenience sampling methods (recruitment flyers, medical provider and staff referrals, community outreach, social media, community listserv posts, and word of mouth referrals) [32, 33]. Individuals interested in participation completed a brief screening survey in-person or by phone. Individuals were eligible if they met the following criteria: age 21 to 64 years [34]; assigned a female sex at birth, now with a masculine spectrum gender identity; have a cervix; have been sexually active within the past 3 years (sexual partner(s) of any gender); able to speak and understand English; and willing and able to provide verbal informed consent. Individuals were provided with a $100 for study participation.

The Trans Masculine Sexual Health Collaborative at Fenway Health, a federally-qualified community health center that serves the lesbian, gay, bisexual, and transgender (LGBT) community in Boston, Massachusetts, conducted the study [35]. A Community and Provider Task Force comprised of 10 individuals was convened to provide guidance to the investigative team, specifically advising the team on methods and ensuring that all aspects of the study (e.g., design, instruments, protocols, procedures, recruitment, branding, website, implementation, interpretation and dissemination of findings) were gender-affirming.

### Procedures

First, participants provided verbal consent (to ease paperwork burden) to participate in the study, as approved by the Institutional Review Board (IRB) at Fenway Health. Next, participants completed a self-administered quantitative survey via an electronic tablet which included socio-demographics, resilience and social-support, discrimination and violence, and mental health. Following the survey, participants completed a clinical visit, followed by an exit interview, both of which are described elsewhere [36]. All study activities were approved by the IRB.

### Measures

#### Independent variables

##### Socio-demographics

Age was assessed continuously in years. Participants were asked to check all that apply regarding their race/ethnicity: White; American Indian/Alaskan Native; Asian; Native Hawaiian or other Pacific Islander; Black/African American; other race/ethnicity. Participants reporting more than one race/ethnicity were coded as such. Participants with a race/ethnicity other than White were coded as being a person of color (POC) to maximize sample size for analysis. Participants were asked whether they were Hispanic or Latino. Additionally, participants were asked to indicate their current gender identity. Participants who identified as a man, male, transgender man, female-to-male (FtM), trans man, man of transgender experience, or trans masculine were coded as having a binary gender identity. Participants who identified as genderqueer, gender non-conforming, non-binary, or another non-binary gender identity were coded as non-binary.

Participants reported the highest level of education they had completed; responses were dichotomized as high school degree or equivalent (i.e., GED, high school diploma, trade school certificate) or some college or more (i.e., some college, Associate’s degree, undergraduate bachelor’s degree, some graduate school, or graduate degree). Participants were asked if they were unemployed versus employed (either full or part time). Annual household income was assessed continuously and coded as low ($32,000 or less, representing 300% of the 2013 federal poverty level) or over $32,000 [37]. Participants also had the option to indicate “don’t know” or “prefer not to answer” when reporting their annual household income. Participants were also asked their current relationship status and could check all apply to the following responses: single; partnered; civil union; married; separated; divorced; widowed; other. Participants who indicated that they were partnered, in a civil union, married, or in another committed relationship, and were not single, were coded as yes, partnered, otherwise no. Unstable housing was assessed by asking participants if they had been homeless or unstably housed in the past 12 months (yes/no). Participants were also asked if they had trouble accessing gender-affirming care in the past 12 months (yes/no).

##### Resilience, social support, and acceptance

Resilience was assessed using 4 items from the Brief Resilience Scale [29, 38]. This scale assesses ability to recover from challenging life events. Item responses are on a scale from 1 = strongly disagree to 5 = strongly agree and included items such as “I tend to bounce back quickly after hard times.” Appropriate item responses were reverse scored and averaged to derive a single scale score (α = 0.86). Higher scores indicated greater ability to recover from stress (i.e., greater resilience).

Social support was measured using an abbreviated version of the Medical Outcomes Study (MOS) Social Support Survey first developed to assess social support in patients with chronic illness [39]. The scale contained 4 items each representing a domain of social support (α = 0.87). Specifically, participants were asked how often, on a scale from 1: None of the time to 5: All of the time, someone is available: To help with daily chores if you are sick? (domain: tangible support); To understand your problems? (domain: emotional/informational support); To get together with you for relaxation (domain: positive social interaction); and To love you and make you feel wanted? (domain: affectionate support). Scores were summed and transformed to be on a scale from 0 to 100, as is standard practice for this measure, with higher scores indicating greater social support [39].

Self-Acceptance was assessed with a single item taken from the Rosenberg Self-Esteem Scale by asking participants, on a scale from 1: No acceptance to 10: Complete acceptance, “I take a positive attitude toward myself” [40, 41]. Transgender Acceptance was assessed with a single item by asking participants, on a scale from 1: No acceptance to 10: Complete acceptance, how much they believe they are accepted by other transgender people.

##### Discrimination & Violence

Intimate partner violence (IPV) was measured using 11 yes/no items assessing whether participants had experienced physical IPV (e.g., whether a partner had ever pressured, physically forced, threatened or blackmailed participants to have sex when they did not want) [42]; sexual IPV (e.g., whether a partner had ever hit, slapped, pushed, shoved, kicked, beat up, choked, burned, used a weapon, or thrown something at a participant in their lifetime) [42]; transgender-related IPV (e.g., whether a partner ever threatened to out them, destroyed their gender affirming treatments) in their lifetime and the past 12 months [43]. Participants experiencing one or more form of IPV were coded as experiencing IPV.

Everyday Discrimination was assessed with the 11-item Everyday Discrimination Scale, which assesses the frequency of participants’ experiences of everyday discrimination in the past 12 months on a Likert scale ranging from 0 = never to 4 = very often [23, 44, 45]. Sample items include: “You have been treated with less courtesy than other people;” “You have received poorer service than other people at restaurants or stores” [44, 45]. Items were summed, and scores ranged from 0 to 39, with higher scores indicating higher levels of everyday discrimination experiences (α = 0.93).

#### Dependent variables

##### PTSD

PTSD symptoms were assessed with a four-item screening scale designed for primary care settings, the Primary Care – PTSD (PC-PTSD) [46]. Participants were asked: “In your life, have you ever had any experience that was so frightening, horrible, or upsetting that, in the past month you: (1) Have had nightmares about it or thought about it when you did not want to? (2) Tried hard not to think about it or went out of your way to avoid situations that reminded you of it? (3) Were constantly on guard, watchful, or easily startled? (4) Felt numb or detached from others, activities, or your surroundings?” Participants responded to each item using binary (yes/no) responses. The response items were summed (α = 0.87) and dichotomized based on a clinical cutoff score of two or more events, validated to ICD-9 PTSD diagnosis or presence of PTSD treatment visit [46].

##### Depression

Participants completed the 18-item Brief Symptom Inventory [47, 48]. Participants were asked to indicate, from 0 (not at all) to 4 (extremely), how much they felt distressed by six symptoms in the past seven days including “feeling lonely” or “feelings of worthlessness” (α = 0.83) [47]. The six items were summed and standardized using T scores (mean of 50 and a standard deviation of 10). Scores are interpreted by comparison to age-appropriate norms. Raw scores are converted to T scores using tables provided in the BSI manual, and T scores of 63 or higher indicated a positive case for depression [47]. This tool has been used in transgender samples previously [30].

##### Anxiety

Participants completed the 18-item Brief Symptom Inventory. Participants were asked to indicate, from 0 (not at all) to 4 (extremely), how much they felt distressed by six symptoms in the past seven days including “feeling fearful” or “feeling tense of cleaned up” (α = 0.88). The six items were summed and standardized using T scores (mean of 50 and a standard deviation of 10). A score of 63 or higher indicated a positive case for anxiety. This tool has also been used in samples of transgender populations [30].

##### Non-suicidal Self-injury (NSSI)

Participants were asked whether they had engaged in self-injurious behavior (e.g., burning, cutting, severe scratching, hitting) without lethal intent in the past 12 months. This item is based on the Self-Injury Questionnaire, which has also been used in transgender samples previously [15–17, 49].

### Data analysis

Statistical analyses of quantitative survey data were conducted in SAS 9.4. Means and frequencies were calculated to describe participant characteristics and assess missingness. Bivariate and multivariable logistic regression models were fit to examine the association between participant characteristics, risk and protective factors, and four mental health status: 1) PTSD; 2) depression; 3) anxiety; and 4) NSSI. Participants reporting “don’t know” or “prefer not to answer” were treated as missing and excluded. Missing data ranged from 1% (*n* = 148) for depression and 11% (*n* = 134) for anxiety. Independent variables significant at *p* < 0.10 in bivariate analyses were included in the multivariable models. Backward selection was used for the multivariable models with significance determined at the *p* < 0.05 level. Area under the curve statistics were calculated to determine the overall predictive validity of the multivariable model. AUC statistics were evaluated based on the following criteria: outstanding (> = .90); excellent (.80–.89); or acceptable (.70–.79) predictive validity [50].

## Results

### Descriptive characteristics

TM adults in the sample had a mean age of 27.5 years (range 21 to 50 years; SD = 5.7), with the majority (72.0%) of participants being between the ages of 21 and 30. Of TM adults in the sample, the majority were White (74.7%), non-Hispanic/Latino (88.7%), and had a binary gender identity (76.7%). The majority had completed some college or more (90.7%) and were employed (76.7%). Almost half (49.3%) of participants had an annual household income of $32,000 or less. The majority (63.3%) were in a relationship (partnered), 12.7% reported unstable housing in the past year, and nearly a quarter (24.7%) had trouble accessing gender affirming care in the past 12 months.

The mean score for resilience was 13.0 (range 10–36; SD = 3.3), 14.8 (range 10–36: SD = 3.9) for social support, 7.7 (range 1–10; SD = 1.4) for self-acceptance, and 7.6 (range 1–10: SD = 2.0) for acceptance by other transgender people. More than two-thirds of participants had experienced intimate partner violence in their lifetime (66.4%) and 14% had experienced some form of violence in the past 12 months. Of the 95 participants in a relationship, 49.5% had experienced IPV in their lifetime, while 9.5% had experienced IPV in the past 12 months. The mean score for everyday discrimination in the past 12-months was 12.9 (range 0–39: SD = 8.8).

Regarding mental health status, 42.2% had PTSD based on past 30-days symptoms; 25.7% had depression based on past 7-day symptoms; 31.1% had anxiety based on past 7-day symptoms; and 31.3% had engaged in NSSI within the past 12-months. Overall, exploring recent mental health comorbidity, 56.7% of the sample had one or more recent mental health problem, 24.0% had one condition, 14.7% had two conditions, 13.3% had three conditions, and 4.7% endorsed all four (recent PTSD, depression, anxiety, and NSSI) Table 1.

**Table 1:** Socio-demographics of a sample of trans masculine adults (*N* = 150)

SOCIO-DEMOGRAPHICS		
Age, continuous	Mean	SD
Range: 21–50 Years	27.5	5.7
Age, categorical	**N**	**%**
21–24	47	31.3
25–29	61	40.7
30–34	27	18
35–39	10	6.7
40–50	5	3.3
Race/Ethnicity		
White	112	74.7
Person of Color	38	25.3
American Indian or Alaska Native	0	0.0
Asian	9	6.0
Native Hawaiian or Pacific Islander	1	0.7
Black or African American	4	2.7
More than one race	24	16.0
Hispanic/Latino		
Hispanic or Latino	14	9.3
Not Hispanic or Latino	133	88.7
Unknown or Not Reported	3	2.0
Gender Identity		
Binary	115	76.7
Non-Binary	35	23.3
Educational Attainment		
High school degree or equivalent	14	9.3
Some college or more	136	90.7
Employment Status - Current (n = 146)		
Unemployed	34	23.3
Employed	112	76.7
Annual Household Income		
$32,000 or less	74	49.3
> $32,000	60	40.0
Don’t know	13	8.7
Prefer not to answer	3	2.0
Partnered - Current		
No	55	36.7
Yes	95	63.3
Unstably Housed – Past 12 Months		
No	131	87.3
Yes	19	12.7
Problems Accessing Gender-Affirming Care - Past 12 Months		
No	113	75.3
Yes	37	24.7
RESILIENCE & ACCEPTANCE		
Resilience Score (n = 148)	Mean	SD
Range (10–36)	13.0	3.3
Social Support Score (*n* = 149)		
Range (10–36)	14.8	3.9
Self-Acceptance (n = 146)		
Range (1–10)	7.7	1.4
Acceptance - Other Trans People (*n* = 146)		
Range (1–10)	7.6	2.0
DISCRIMINATION & VIOLENCE		
Any IPV - Lifetime (n = 149)*	**N**	**%**
No	50.0	33.6
Yes	99.0	66.4
Everyday Discrimination	Mean	SD
Range (0–39)	12.9	8.8
MENTAL HEALTH		
PTSD - Past 30 Days (n = 147)		
No	85	57.8
Yes	62	42.2
Depressed - Past 7 Days (n = 148)		
No	110	74.3
Yes	38	25.7
Anxiety - Past 7 Days (*n* = 148)		
No	102	68.9
Yes	46	31.1
NSSI - Past 12 Months (*n* = 147)		
No	102	69.4
Yes	46	31.3
COMORBIDITY OF RECENT MENTAL HEALTH PROBLEMS		
One or more	85	56.7
One Condition	36	24.0
PTSD Only	22	14.7
Depression Only	6	4.0
Anxiety Only	4	2.7
NSSI Only	4	2.7
Two Conditions	22	14.7
Depression & NSSI	1	0.7
Depression & Anxiety	7	4.7
PTSD & Depression	2	1.3
PTSD & NSSI	3	2.0
PTSD & Anxiety	9	6.0
Three Conditions	20	13.3
Depression, Anxiety, & NSSI	1	0.7
PTSD, Depression & NSSI	1	0.7
PTSD, Anxiety & NSSI	5	3.3
PTSD, Depression & Anxiety	13	8.7
Four Conditions		
PTSD, Depression, Anxiety & NSSI	7	4.7

Note**:** *PTSD* post-traumatic stress disorder, *IPV* Intimate partner violence, *NSSI* non-suicidal self-injury

### Mental health status

#### PTSD

In the first multivariable model, being unemployed (referent = employed; aOR = 2.76; 95% CI = 1.04–7.29; *p* = 0.04), lifetime IPV (referent = no IPV; aOR = 3.08; 95% CI = 1.26–7.53; *p* = 0.01), and higher levels of past 12-month everyday discrimination (aOR = 1.07; 95% CI = 1.02–1.12; p = 0.01) were each significantly associated with an increased odds of PTSD, while having a partner (referent = no partner; aOR = 0.38; 95% CI = 0.17–0.88; *p* = 0.02) was associated with the reduced odds of PTSD. The predictive validity of the model was shown to be acceptable (AUC = 0.75).

#### Depression

In the second model, having high school degree or equivalent (referent = some college or more; aOR = 4.71; 95% CI = 1.06–20.96; *p* = 0.04) and higher levels of past 12-month everyday discrimination (aOR = 1.08; 95% CI = 1.03–1.13; *p* = 0.002) were each associated with the increased odds of being depressed. Conversely, higher resilience scores were associated with the reduced odds of being depressed (aOR = 0.81; 95% CI = 0.71–0.93; *p* = 0.003). The predictive validity of the model was shown to be acceptable (AUC = 0.77).

#### Anxiety

In the third multivariable model, having a household income of $32,000 a year or less (referent = more than $32,000; aOR = 3.95; 95% CI = 1.61–9.71; p = 0.003) and higher levels of past 12-month everyday discrimination (aOR = 1.09; 95% CI = 1.03–1.15; p = 0.003) were each associated with the increased odds of anxiety. Higher resilience scores were associated with reduced odds of anxiety (aOR = 0.77; 95% CI = 0.66–0.90; *p* < 0.001) in this model. The predictive validity of the model was shown to be excellent (AUC = 0.82).

#### NSSI

In the fourth multivariable model, higher past 12-month everyday discrimination scores (aOR = 1.06; 95% CI = 1.01–1.13; *p* = 0.03) were associated with an increased odds of past 12-month NSSI, while older age (aOR = 0.85; 95% CI = 0.74–0.98; *p* = 0.02) and higher levels of resilience (aOR = 0.78; 95% CI = 0.66–0.91; *p* = 0.002) were each associated with a reduced odds of NSSI. The predictive validity of the model was shown to be excellent (AUC = 0.81) Table 2.

**Table 2:** Bivariate and multivariable associations between socio-demographics, resilience, support, acceptance, violence, and discrimination and mental health and substance use status in a sample of trans masculine adults (N = 150)

	PTSD						BSI - DEPRESSED						BSI - ANXIETY						NSSI - PAST 12 MONTHS					
	Bivariate			Multivariable			Bivariate			Multivariable			Bivariate			Multivariable			Bivariate			**Multivariable**		
	OR	95% CI	P-Value	aOR	95% CI	P-Value	OR	95% CI	P-Value	aOR	95% CI	P-Value	OR	95% CI	P-Value	aOR	95% CI	P-Value	OR	95% CI	P-Value	aOR	95% CI	P-Value
Age																								
Continuous	1.00	0.95–1.06	0.94				0.91	0.84–0.99	**0.03**	**†**	**†**	**†**	0.97	0.91–1.04	0.35				0.82	0.71–0.94	**0.004**	0.85	0.74–0.98	**0.02**
Race																								
White	1.00	–	–	–	–	–	1.00	–	–	–	–	–	1.00	–	–	–	–	–	1.00	–	–	–	–	–
Person of color	0.97	0.45–2.08	0.94	–	–	–	0.75	0.31–1.81	0.52	–	–	–	0.77	0.34–1.77	0.54	–	–	–	1.55	0.58–4.15	0.39	–	–	–
Gender Identity																								
Binary	1.00	–	–	–	–	–	1.00	–	–	1.00	–	–	1.00	–	–	1.00	–	–	1.00	–	–	–	–	–
Nonbinary	1.76	0.82–3.82	0.15	–	–	–	2.20	0.97–5.01	0.06	**†**	**†**	**†**	2.11	0.96–4.67	0.06	**†**	**†**	**†**	1.69	0.63–4.57	0.30	–	–	–
Educational Attainment																								
High school or equivalent	0.38	0.10–1.45	0.16	–	–	–	3.25	0.98–10.78	0.05	4.71	1.06–20.96	**0.04**	1.12	0.32–3.92	0.86	–	–	–	1.15	0.23–5.64	0.86	–	–	–
Some college or more	1.00	–	–	–	–	–	1.00	–	–	1.00	–	–	1.00	–	–	–	–	–	1.00	–	–	–	–	–
Employment Status																								
Employed	1.00	–	–	1.00	–	–	1.00	–	–	–	–	–	1.00	–	–	–	–	–	1.00	–	–	–	–	–
Unemployed	2.99	1.33–6.70	**0.01**	2.76	1.04–7.29	**0.04**	1.02	0.43–2.43	0.97	–	–	–	1.24	0.55–2.78	0.61	–	–	–	1.26	0.45–3.52	0.66	–	–	–
Annual Household Income																								
$32,000 or less	2.58	1.27–5.25	**0.01**	†	†	**†**	2.53	1.14–5.62	**0.02**	**†**	**†**	**†**	4.11	1.87–9.00	**< 0.001**	3.95	1.61–9.71	**0.003**	1.89	0.71–5.06	0.20	–	–	–
> $32,000	1.00	–	–	1.00	–	–	1.00	–	–	1.00	–	–	1.00	–	–	1.00	–	–	1.00	–	–	–	–	–
Partnered																								
No	1.00	–	–	1.00	–	–	1.00	–	–	–	–	–	1.00	–	–	1.00	–	–	1.00	–	–	–	–	–
Yes	0.45	0.23–0.89	**0.02**	0.38	0.17–0.88	**0.02**	0.54	0.26–1.15	0.11	–	–	–	0.50	0.24–1.02	*0.06*	**†**	**†**	**†**	1.25	0.47–3.29	0.65	–	–	–
Unstably Housed - Past 12 Months																								
No	1.00	–	–	1.00	–	–	1.00	–	–	1.00	–	–	1.00	–	–	1.00	–	–	1.00	–	–	1.00	–	–
Yes	4.24	1.43–12.63	**0.01**	†	†	**†**	2.67	0.97–7.36	0.06	**†**	**†**	**†**	2.51	0.93–6.83	*0.07*	**†**	**†**	**†**	3.53	1.16–10.73	**0.03**	**†**	**†**	**†**
Problems Accessing Gender-Affirming Care - Past 12 Months																								
No	1.00	–	–	1.00	–	–	1.00	–	–	–	–	–	1.00	–	–	–	–	–	1.00	–	–	–	–	–
Yes	2.39	1.11–5.15	**0.03**	†	†	**†**	0.91	0.38–2.15	0.83	–	–	–	1.76	0.81–3.82	0.15	–	–	–	1.89	0.72–4.95	0.19	–	–	–
RESILIENCE, SUPPORT & ACCEPTANCE																								
Resilience Score (*n* = 148)																								
Continuous	0.90	0.81–0.99	**0.04**	†	†	**†**	0.80	0.71–0.91	**< 0.001**	0.81	0.71–0.93	**0.003**	0.79	0.70–0.89	**< 0.001**	0.77	0.66–0.90	**< 0.001**	0.74	0.64–0.87	**< 0.001**	0.78	0.66–0.91	**0.002**
Social Support Score (*n* = 149)																								
Continuous	0.91	0.83–0.99	**0.02**	†	†	**†**	0.91	0.83–1.00	0.05	**†**	**†**	**†**	0.88	0.80–0.97	**0.01**	–	–	–	0.97	0.86–1.08	0.55	–	–	–
Self-Acceptance (n = 146)																**†**	**†**	**†**						
Continuous	0.77	0.59–1.00	0.05	†	†	**†**	0.69	0.52–0.91	**0.01**	**†**	**†**	**†**	0.80	0.62–1.03	0.08	**†**	**†**	**†**	0.81	0.59–1.10	0.18	–	–	–
Acceptance - Other Trans People (*n* = 146)																								
Continuous	0.80	0.67–0.95	**0.01**	†	**†**	**†**	0.78	0.65–0.93	**0.01**	**†**	**†**	**†**	0.79	0.66–0.94	**0.01**	**†**	**†**	**†**	0.92	0.74–1.14	0.44	–	–	–
DISCRIMINATION & VIOLENCE																								
Any IPV - Lifetime																								
No	1.00	–	–	1.00	–	–	1.00	–	–	1.00	–	–	1.00	–	–	–	–	–	1.00	–	–	–	–	–
Yes	3.75	1.72–8.17	**< 0.001**	3.08	1.26–7.53	**0.01**	2.74	1.11–6.77	**0.03**	**†**	**†**	**†**	2.23	1.00–4.99	0.05	**†**	**†**	**†**	0.85	0.33–2.20	0.74	–	–	–
Everyday Discrimination - Past 12 Months																								
Continuous	1.09	1.05–1.14	**< 0.001**	1.07	1.02–1.12	**0.01**	1.09	1.04–1.14	**< 0.001**	1.08	1.03–1.13	**0.002**	1.11	1.06–1.17	**< 0.001**	1.09	1.03–1.15	**0.003**	1.08	1.02–1.13	**0.01**	1.06	1.01–1.13	**0.03**

Note: Bolded p-values = significant a *p* < 0.05. Multivariable models included any variable with an association <= 0.10 and used backward selection. Final models 1 to 11% missing: PTSD (*n* = 137); Depressed (*n* = 148); Anxiety (*n* = 134); and Self Harm (*n* = 147)

† Included in multivariable model and ultimately excluded via backward selection

*PTSD* post-traumatic stress disorder, *IPV* Intimate partner violence, *NSSI* non-suicidal self-injury

Predictive validity of model based on Area Under the Curve: Model 1 = 0.75 (acceptable); Model 2 = 0.77 (acceptable); Model 3 = 0.82 (excellent); Model 4 = 0.81 (excellent)

## Discussion

This study documents mental health burden in TM adults and illuminates risk and protective factors for mental health status in this subgroup of transgender people [51]. Reported mental health status in our data reflect high prevalence of mental health morbidity, including co-morbidity, as previously documented elsewhere [6–13, 30]. Further, consistent with previous research among diverse transgender people [6, 8, 20, 27, 52], low socioeconomic status, lack of college education, discrimination, and violence were risk factors for poor mental health, while resilience, older age, and being in a committed relationship were protective in this sample of TM adults. Future research on the implementation of social supports to mitigate the aforementioned risk factors and how to best bolster recovery capacity, or resiliency, among at-risk TM adults is crucial.

Key socio-demographic factors emerged as risk factors for poor mental health in this community sample of TM adults. Having a low income and limited educational attainment were associated with the heightened odds of depression and anxiety, respectively. Past studies have documented the relationship of limited education and depression in transgender women [8], while low-income has been positively linked to anxiety in TM adults [6]. A recent review of stigma among gender minority people found most transgender victims of violence were low-income [53]. Structural stigma can contribute to low socioeconomic status via employment and educational discrimination for transgender individuals. Lack of access to essential resources can be stressful and may therefore contribute to poor mental health among transgender individuals [7, 8, 20, 27]. Findings from the present study further elucidate the relationship between low-income, limited education, and poor mental health in TM adults specifically [20]. Future longitudinal research will be important to explore the interplay and temporality between structural stigma, socioeconomic status, and poor mental health in TM.

Stigma may also shape poor mental health through interpersonal stigma, including everyday discrimination and IPV. Consistent with prior research exploring the relationship between discrimination, IPV and PTSD in a sample of both TM and TF adults, the present study found that both past 12-month everyday discrimination and lifetime IPV were associated with elevated risk for PTSD [24]. However, unique to this study, everyday discrimination was the only risk factor found to be associated with all four mental health statuses (i.e., PTSD, depression, anxiety, and NSSI), even after controlling for socio-demographics, protective factors, and IPV. Numerous studies have documented the damaging relationship between discrimination and poor mental health (e.g., mood and anxiety disorders, PTSD, suicidality, and substance use disorders) in diverse transgender samples [20, 24, 27, 52, 54–57]. To our knowledge, this is the first study to demonstrate the link between discrimination and multiple forms of poor mental health in TM adults specifically.

Studying the lived experiences of TM in particular is key to address risk factors, promote protective factors, and screen for and treat mental health symptoms and conditions. In addition to routine assessment of lifetime partner violence, recent discrimination has a powerful association with current mental health and should be assessed clinically as a risk factor for mental health morbidity among TM adults. Additional research is needed to explore the influence of discrimination and IPV experienced by TM adults including frequency, severity, and perpetrator gender over time. Previous studies focused on sexual minority (LGB) people have found that state-level policies (i.e., banning hate crimes, employment discrimination, and bullying) can improve mental health status [58, 59]. Such findings can potentially be applied to gender minority populations and considered for future advocacy and policymaking efforts.

Several interpersonal and individual factors emerged as protective for mental health in this sample of TM adults. Specifically, being in a relationship was independently associated with a lower odds of PTSD. Although a lifetime history of violence in a relationship was associated with worse mental health in this study, being in a current committed relationship appeared to be beneficial for mental health. Additionally, whereas almost half of participants currently in a relationship endorsed a lifetime history of IPV, only a tenth of them endorsed IPV in the past year. This finding could be suggestive of the mental health benefits associated with social support, as well as feeling loved by a partner [55, 60–63]. Additional mixed methods research is a needed to explore what aspects of being in relationships are helpful for the mental health of TM adults.

Additionally, two individual factors, older age and personal resilience, were found to be protective against poor mental health in this study. The relationship between younger age and NSSI has been previously documented among transgender adults, although this is the first study to our knowledge to demonstrate this association among TM adults specifically [64]. Increasing social support and promoting resilience can serve as important tools to combat stigma, which is a known risk factor for mental illness in gender minority adults [20, 28]. This finding extends previous research, documenting the important relationship between resiliency and improved mental health in TM individuals. Future research efforts should explore how resiliency can be leveraged in interventions to improve the mental health of TM, especially in the face of known risk factors for poor mental health such as discrimination, violence, and other forms of stigma.

## Limitations

Several limitations should be noted. The cross-sectional study design does not allow for causal inference regarding the relationships between the independent variables and the mental health dependent variables. However, mental health variables were selected to use the most recently measured statuses (e.g., past-week depression) in an attempt to establish a temporal ordering of independent variables and outcomes. Additionally, the majority of this Boston-area sample was comprised of young and white TM, which may limit the generalizability of results to TM people of color, older TM adults, and those in other areas of the U.S. In addition, data analyzed were from a biobehavioral study of TM sexual health, which enrolled TM adults with a cervix eligible for cervical cancer screenings. Thus, findings may not generalize to TM adults who have had gender confirmation surgery or who may be unwilling to undergo clinical procedures for cervical cancer screening. For example, TM adults who lack access to transportation, or who may feel uncomfortable or “othered” in health care settings may not have been adequately sampled. Moreover, all measures were based on self-report, which may have contributed to social desirability bias for the sensitive data collected, although the use of computer-assisted personal interviewing techniques likely minimized this bias. Finally, the mental health statuses were based on validated measures of clinical symptoms rather than clinical diagnoses by a health care provider, which may lead to higher prevalence estimates of poor mental health than studies reporting current or lifetime clinical diagnoses.

## Conclusions

This study found that low income, limited education, discrimination, and IPV were risk factors for poor mental health, while being in a relationship, older age, and personal resilience were protective against poor mental health in a community sample of TM adults. These findings highlight the need for multi-level interventions targeting individual, interpersonal, and structural factors that may be driving poor mental health in this population. Findings pave the way for future public health research and program implementation to promote mental health equity for TM individuals.

## Abbreviations

BRFSS: Behavioral Risk Factors Surveillance System
CDC: Centers for Disease Control and Prevention’s
FtM: Female-to-male
IPV: Intimate partner violence
LGBT: Lesbian, gay, bisexual, transgender
MOS: Medical Outcomes Study
NSSI: Non-suicidal self-injury
POC: People of color
PTSD: Post-traumatic stress disorder
TF: Trans-feminine
TM: Trans-masculine
U.S.: United States
