# Correction to: Risk and protective factors for mental health morbidity in a community sample of female-to-male trans-masculine adults

**DOI:** 10.1186/s12888-019-2028-4

**Published:** 2019-01-28

**Authors:** Michal J. McDowell, Jaclyn M. W. Hughto, Sari L. Reisner

**Affiliations:** 1 Harvard Medical School, 300 Longwood Ave, Boston, MA USA; 2 Department of Epidemiology, Brown University School of Public Health, Providence, RI USA; 3 Fenway Health, The Fenway Institute, Boston, MA USA; 4 Department of Epidemiology, Harvard T.H. Chan School of Public Health, Boston, MA USA; 5 Division of General Pediatrics, Boston Children’s Hospital, Boston, MA USA

**Correction to: BMC Psychiatry**

**https://doi.org/10.1186/s12888-018-2008-0**

Following publication of the original article [1], we have been notified of the below corrections.

- NSSI prevalence: The sample prevalence (31.1%) was accidentally reported for both anxiety and NSSI. The prevalence of NSSI was 15.0% had engaged within the past 12-months.
- Annual household income: Low income was written as $32,000 instead of $30,000. Annual household income was assessed continuously and coded as low (less than $30,000 - representing ~ 260% of the 2013 federal poverty level, $30,000 or more than $30,000).
- The second author’s name should be written as: Jaclyn M.W. Hughto
